# Crosstalk between ROCK1 and PYROXD1 regulates CAFs activation and promotes laryngeal squamous cell carcinoma metastasis

**DOI:** 10.1007/s12672-022-00578-y

**Published:** 2022-11-05

**Authors:** Liyun Yang, Peipei Qiao, Jianwei Zhang, Xiaoping Chen, An Hu, Shuixian Huang

**Affiliations:** Department of Otolaryngology, Gongli Hospital, The Second Military Medical University, Shanghai, 200135 China

**Keywords:** PYROXD1, ROCK1, CAFs activation, Metastasis, LSCC

## Abstract

We previously found that the Rho-associated kinase 1 (ROCK1) activated Cancer-associated fibroblasts (CAFs) to promote LSCC metastasis. Accumulating evidence indicates that pyridine nucleotide-disulfide oxidoreductase domain 1 (PYROXD1) is an oncogene; however, the crosstalk between ROCK1 and PYROXD1 in LSCC metastasis remains largely unknown. Here, we found that ROCK1 could target PYROXD1. The knockdown of ROCK1 expression reduces the expression of PYROXD1, while the knockdown of PYROXD1 expression did not alter the expression of ROCK1 indicating that ROCK1 is upstream of PYROXD1. Further, LSCC cells cocultured with PYROXD1 knocked-down CAFs exhibited lower proliferation, migration, invasion and metastasis abilities. Conversely, LSCC cells cocultured with PYROXD1-overexpressing CAFs showed opposite results. In conclusion, the crosstalk between ROCK1 and PYROXD1 regulated CAFs activation and promoted LSCC metastasis.

## Introduction

Laryngeal squamous cell carcinoma (LSCC) is one of the most common primary malignant cancers and also is the most common pathological subtype of laryngeal cancer [1]. Studies have reported that 177,422 people were diagnosed with LSCC with a mortality rate of 46.7% [2]. The incidence of LSCC in China is four times as high as that in the United States, and the mortality rate is also markedly higher [3]. Despite continuous improvement in LSCC treatment, its 5-year survival rate remains mediocre, at < 50% [4]. Therefore, elucidating the underlying mechanisms of LSCC metastasis is vital for identifying novel and more efficient therapies for LSCC.

Rho-associated kinase 1 (ROCK1) is a classic serine/threonine protein kinase of the A, G, and C protein kinase families [5]. ROCK1 regulates cellular processes such as actomyosin contractility, focal adhesion assembly, cytokinesis, and cell proliferation [6]. Accumulating evidence suggests that dysfunction in ROCK1 can lead to various cancers, including bladder [7], breast [8], ovarian [9], gastric [10], colon [11] and ovarian cancers [12]. Similarly, our previous study showed that ROCK1 was highly expressed in human LSCC tissues, and high expression of ROCK1 corelated with advanced stage and poor LSCC survival. However, the mechanisms by which ROCK1 promotes LSCC metastasis remain largely unclear.

It is well known that tumor metastasis is not only closely correlated with the properties of tumor cells, but also with tumor stroma [13]. Cancer-associated fibroblasts (CAFs) are the major tumor stromal cells. CAFs actively communicate with cancer cells, and in many solid tumors contribute to tumor progression through secretion of growth factors, hormones and cytokines [14–16]. Therefore, we sought to investigate the crosstalk between ROCK1 and PYROXD1 to regulate CAFs activation. And explore the role of ROCK1 and PYROXD1 in metastasis of LSCC.

In this present study, we found that ROCK1 interacted with PYROXD1 and that the knockdown of ROCK1 expression by siRNA reduced the expression of PYROXD1, while the knockdown of PYROXD1 expression did not alter the expression of ROCK1 indicating that ROCK1 is upstream of PYROXD1. Additional experiments showed that LSCC cells cocultured with PYROXD1-knockdown CAFs had reduced proliferation, migration, invasion and metastasis abilities. Conversely, LSCC cells cocultured with PYROXD1-overexpressing CAFs showed opposite results. Thus, the crosstalk between ROCK1 and PYROXD1 regulated CAF activation and promoted the metastasis of LSCC suggesting that the ROCK1 and PYROXD1 as new therapeutic targets for treating LSCC.

## Materials and methods

### Cell culture

LSCC cell lines (TU686 and TU212 cells) were obtained from the Shanghai Institutes from Biological Sciences. LSCC tissues were minced into small pieces and seeded in dishes (10 cm) to isolate CAFs and NFs. The cells were cultured with DMEM supplemented with 10% FBS, and 100 IU/ml penicillin and streptomycin were added to prevent infection. All cells were cultured in a humidified cell incubator with an atmosphere of 5% CO_2_ at room temperature.

### Transfection

CAFs were transfected with PYROXD1 siRNA using a transfection reagent. The si-ROCK1 sequence was 5′-GAAGAAACATTCCCTATTC-3′, and the negative control siRNA (si-NC) sequence was 5′-CGTCAACATGGCTTTCACC-3′ [17]. The PYROXD1 siRNA sequences were GGAUAAUGAUUGUCGGGAA, CGAGGGAAAUCCACGUGUA, ACAUUAAGGUCAUCGAAUC, and CCAUAAAGGAUAACGCCAU. CAFs were transfected with scrambled siRNA to establish the negative control groups [18]. All the three numbers and the power analysis indicating the sufficient three numbers for each experiment.

### Western blotting analysis

The RIPA buffer (Pierce, Rockford, USA) was used to lyse the cells after different treatments. The Proteins (100 μg/sample) were electrophoresed with 10% SDS-PAGE for 2 h. Then, they were transferred to 0.22 µm PVFD membranes (Millipore, MA, USA) and incubated with primary antibodies (anti-ROCK1, anti-PYROXD1, and anti-GAPDH) and a secondary antibody. All the antibodies were obtained from Cell Signaling Technology Company. The proteins were visualized using a chemiluminescence detection system (Amersham Bioscience, Piscataway, NJ, USA).

### Co-IP assay

The manufacturer’s instructions were followed to perform the Co-IP assay. Briefly, immobilized affinity-purified antibodies (10–75 μg) were used in the experimental group, and immobilized anti-IgG was used in the control group. Then, 200–400 μl IP lysate/well was added to 6-well plates. The proteins were extracted from the lysates at 13,000×*g* for 10 min and incubated with immobilized antibodies for 1 day at 4 °C. Then, they were subjected to Western blotting. All the Co-IP-related reagents were purchased from Thermo Scientific Company. The primary antibodies (anti-ROCK1, anti-PYROXD1 and anti-rabbit IgG) were purchased from Cell Signaling Technology Company.

### CCK-8 assay

TU686 and TU212 cells were cocultured with CAF/si-PYROXD1, CAF/si-nc, recPYROXD1 and PBS. Then, 2 × 10^3^ TU686 and TU212 cells/well were seeded into 96-well plates and CCK8 (10 µl) was added to each well. After incubation for 2 h, the OD450 value of every well was measured.

### Wound healing assay

TU686 and TU212 cells were cocultured with CAF/si-PYROXD1, CAF/si-nc, recPYROXD1 and PBS. Then, 1 × 10^6^ TU686 and TU212 cells/well were seeded in 6-well plates and incubated overnight. The cells were scratched with a 20 μl pipette tip and photographed with a high-power microscope (2×) at 0, 1 and 2 days.

### Plate colony formation assay

TU686 and TU212 cells were cocultured with CAF/si-PYROXD1, CAF/si-nc, recPYROXD1 and PBS. Then, 1 × 10^3^ and 2 × 10^3^ TU686 and TU212 cells/well were seeded in 6-well plates. DMEM supplemented with 10% FBS was used to culture the cells for 21 days, after which the cells were washed with PBS twice and stained with crystal violet for half an hour. The colonies formed in each well were then counted.

### Apoptosis assay

TU686 and TU212 cells were cocultured with CAF/si-PYROXD1, CAF/si-nc, recPYROXD1 and PBS. Then, TU686 and TU212 cells were treated with celastrol for 2 days and washed with PBS three times. The cells were resuspended with 1× binding buffer, following which Annexin V staining was performed for 30 min at room temperature. The cells were then double stained with PI at room temperature for half an hour and the percentages of apoptotic cells were quantified by flow cytometry.

### Transwell assay

TU686 and TU212 cells were cocultured with CAF/si-PYROXD1, CAF/si-nc, recPYROXD1 and PBS. Then, 2 × 10^5^ TU686 and TU212 cells were starved for 24 h and plated on coated filters and 600 μl of medium supplemented with 10% FBS was added to the lower chamber. The cells were then counted randomly with a high-power microscope (10×). Diluted Matrigel (BD Biosciences) was coated on the membrane to perform the invasion assay. The other experimental steps were the same as those in the migration assay.

### In vivo metastasis model

The animal care and experiments were approved by the Experimental Animal Ethics Committee of Shanghai Jiao Tong University School of Medicine. Four-week-old male nude mice were injected with 2 × 10^6^ cells (TU686 + CAF/si-PYROXD1, TU212 + CAF/si-PYROXD1, TU686 + CAF/si-nc, TU212 + CAF/si-nc, TU686 and TU212 cells) via the tail vein. The mice were obtained from the Institute of Zoology, Chinese Academy of Sciences. After housing the nude mice for one and a half months, the mice were killed, and their lungs were dissected. Then, their lung metastatic nodules were quantified by H&E staining.

### Statistical analysis

The GraphPad Prism 6 software was used to analyze the data, and the data are presented as the mean ± standard deviation (SD). Data were compared with the t-test and one-way analysis of variance (ANOVA), and P < 0.05 was considered statistically significant.

## Results

### ROCK1 targets PYROXD1 in LSCC cells

**Fig. 1:**
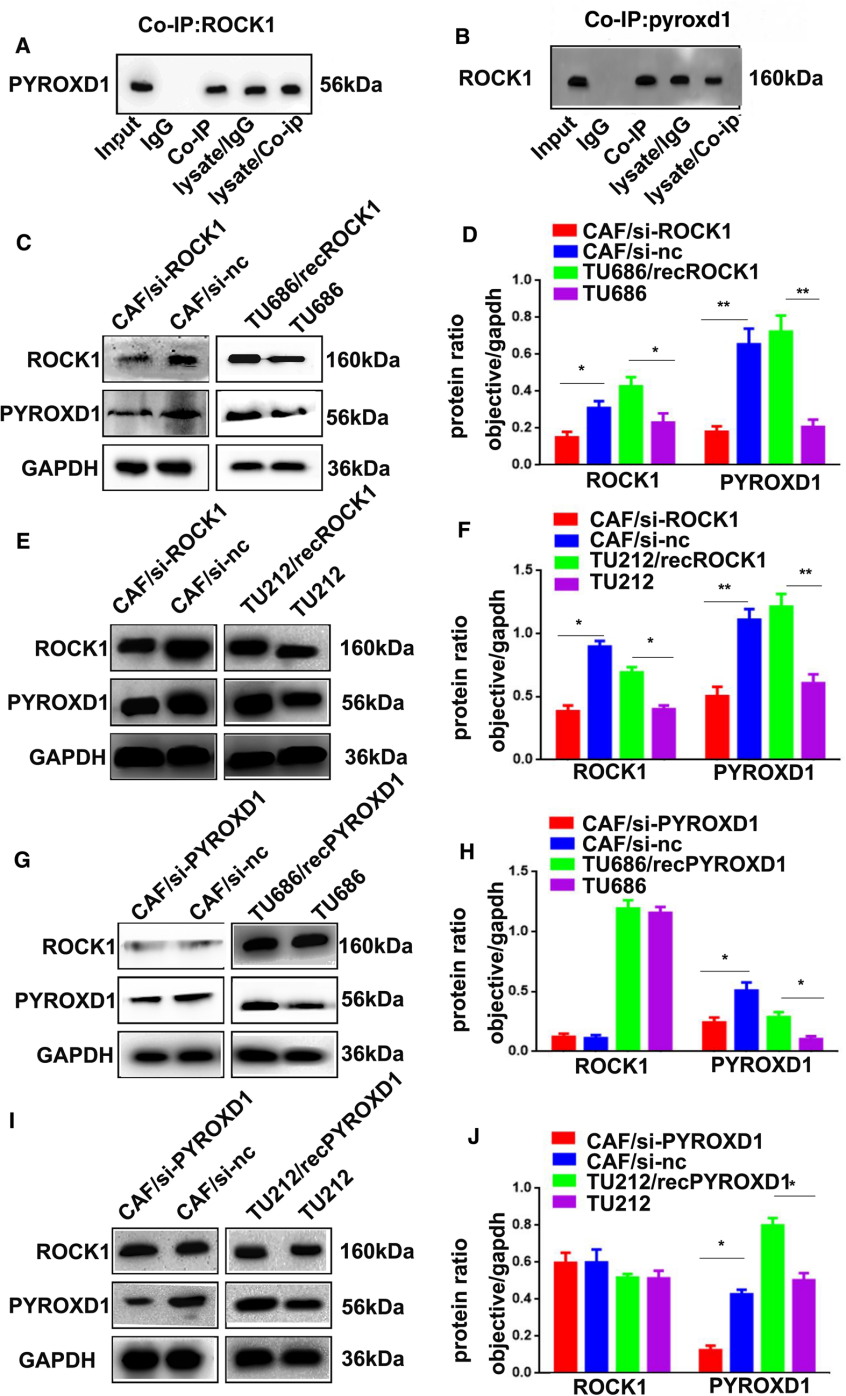
ROCK1 targets PYROXD1 in LSCC cells. **A**, **B** Co-IP was used to examine the relationship between ROCK1 and PYROXD1. **C**, **D** The expression levels of ROCK1, PYROXD1 and GAPDH in CAF/si-ROCK1, CAF/si-nc, U686/recROCK1, TU212/recROCK1, TU686 and TU212 cells were analyzed by Western blotting. **E**, **F** The expression levels of ROCK1, PYROXD1 and GAPDH in CAF/si-PYROXD1, CAF/si-nc, U686/recPYROXD1, TU212/recPYROXD1, TU686 and TU212 cells were analyzed by Western blotting

Our previous study found that the protein kinase ROCK1 played a key role in activating CAFs to promote LSCC metastasis. Accumulating evidence indicates that the oxidoreductase PYROXD1 may act as a positive regulator of tumorigenesis. Therefore, a Co-IP assay was performed to further investigate the relationship between the protein kinase ROCK1 and the oxidoreductase PYROXD1 in LSCC cells. As shown in Fig. 1A, B, ROCK1 targeted PYROXD1. To further determine whether PYROXD1 expression was induced by ROCK1 to accelerate CAF activation, CAFs were transfected with si-ROCK1 and si-PYROXD1 (CAF/si-ROCK1 and CAF/si-PYROXD1 cells) or the negative controls (CAF/si-nc cells). Western blotting results showed that the expression of ROCK1 and PYROXD1 was decreased in the CAF/si-ROCK1 cells (*p < 0.05, **p < 0.01, Fig. 1C–F), the expression of PYROXD1 was decreased in the CAF/si-PYROXD1 cells, while the expression of ROCK1 was not altered (*p < 0.05, Fig. 1G–J). Additionally, TU686 and TU212 cells were treated with purified recombinant ROCK1 protein (TU686/recROCK1 and TU212/recROCK1 cells) and PYROXD1 protein (TU686/recPYROXD1 and TU212/recPYROXD1 cells). Obviously, TU686/recROCK1, TU212/recROCK1, TU686/recPYROXD1 and TU212/recPYROXD1 cells showed higher ROCK1 and PYROXD1 levels than TU686 and TU212 cells (*p < 0.05, **p < 0.01, Fig. 1C–J). The expression of ROCK1 did not change in TU686/recPYROXD1, TU212/recPYROXD1, TU686 or TU212 cells (Fig. 1G–J). These findings indicated that ROCK1 could target the oxidoreductase PYROXD1 in LSCC cells.

### Downregulation of PYROXD1 expression reduces LSCC cell proliferation, migration, and invasion

**Fig. 2:**
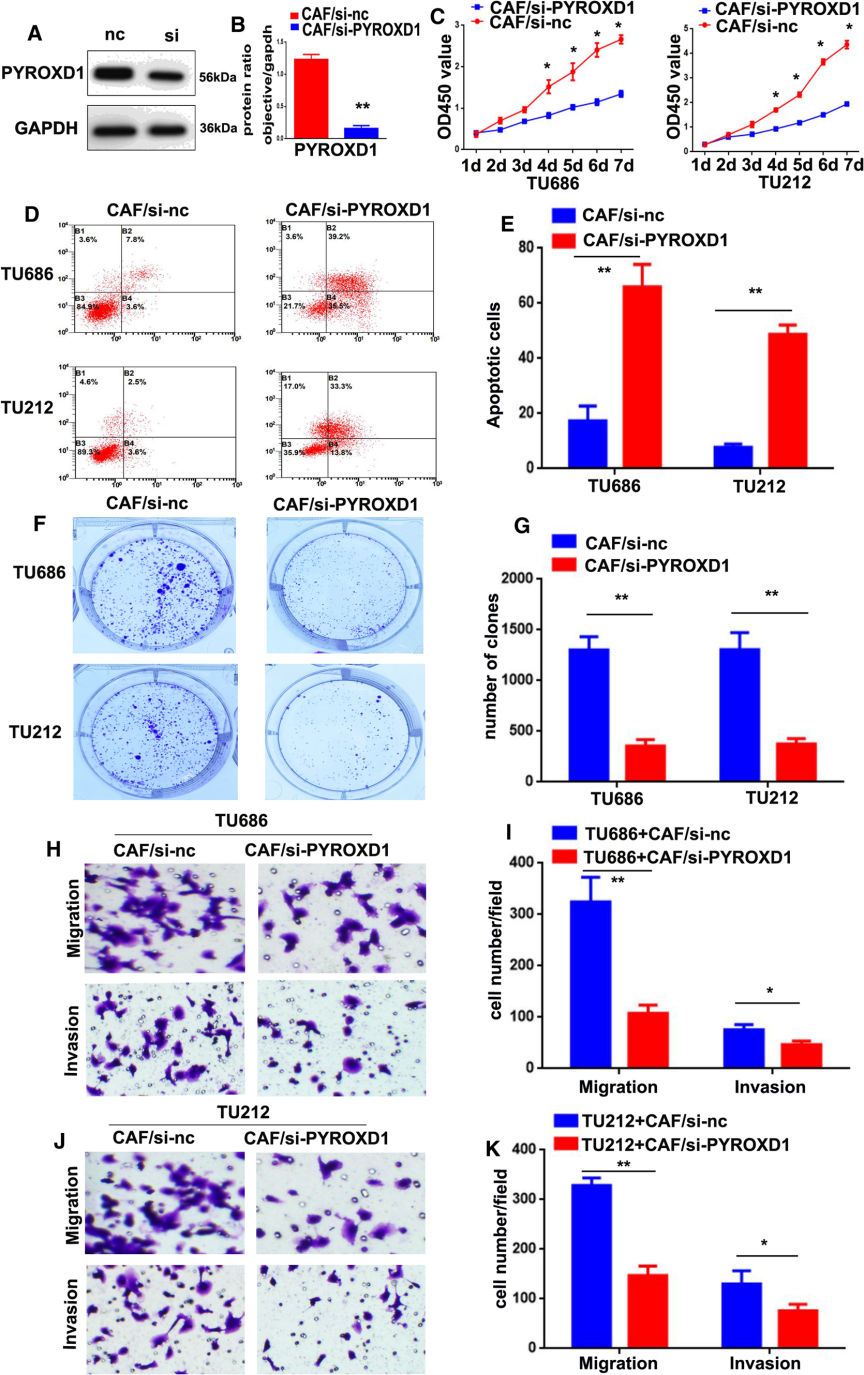
PYROXD1 downregulation reduces the proliferation, migration, and invasion of LSCC cells. **A** PYROXD1 protein expression in CAFs transfected with PYROXD1 siRNA (siPYROXD1) was assessed by Western blotting. **B** Diagram of the in vitro coculture system. **C** Knockdown of PYROXD1 expressed reduced the proliferation of TU686 + CAF/si-PYROXD1 and TU212 + CAF/si-PYROXD1 cells (*P < 0.05). **D**, **E** Knockdown of PYROXD1 expression induced the apoptosis of TU686 + CAF/si-PYROXD1 and TU212 + CAF/si-PYROXD1 cells (**P < 0.01). **F**, **G** Knockdown of PYROXD1 expression decreased the clone formation abilities of TU686 + CAF/si-PYROXD1 and TU212 + CAF/si-PYROXD1 cells (**P < 0.01). **H**–**K** Knockdown of PYROXD1 expression reduced the migration and invasion of TU686 + CAF/si-PYROXD1 and TU212 + CAF/si-PYROXD1 cells (*P < 0.05, **P < 0.01). Migratory and invaded Cells were counted in ten randomly selected microscopic fields. Values are represented as mean ± SD of three independent experiments

After the knockdown of PYROXD1 expression was confirmed (**p < 0.01, Fig. 2A, B), we established an in vitro coculture system, whereby, TU686 and TU212 cells were analyzed by CCK-8, apoptosis, colony formation, migration and invasion assays at indicated time points. As shown in Fig. 2C, the proliferation of both the TU686 + CAF/si-PYROXD1 and TU212 + CAF/si-PYROXD1 groups was lower than that of the TU686 + CAF/si-nc and TU212 + CAF/si-nc groups (*p < 0.05). Flow cytometry results showed that the percentage of apoptotic cells was significantly higher in the TU686 + CAF/si-PYROXD1 (65.9 ± 8.2) and TU212 + CAF/si-PYROXD1 groups (48.7 ± 3.4) than in the TU686 + CAF/si-nc (17.4 ± 5.4) and TU212 + CAF/si-nc groups (7.7 ± 1.2, **P < 0.01, Fig. 2D, E). Colony formation assay was performed to further verify the proliferative effect of PYROXD1, and the results showed that the numbers of colonies in the TU686 + CAF/si-PYROXD1 (356 ± 62.2) and TU212 + CAF/si-PYROXD1 (376 ± 50.5) groups were lower than those in the TU686 + CAF/si-nc (1304 ± 128.5) and TU212 + CAF/si-nc groups (1305 ± 168.9, **P < 0.01, Fig. 2H–K). The results also indicated that the TU686 + CAF/si-nc (324 ± 48.1) and TU212 + CAF/si-nc cells (328 ± 15.2) migrated at higher rates through the Transwell chambers than TU686 + CAF/si-PYROXD1 (107 ± 16.3) and TU212 + CAF/si-PYROXD1 cells (147 ± 19.1, *P < 0.05, **P < 0.01, Fig. 2H–K). Lastly, these results of the invasion assays showed that the TU686 + CAF/si-nc (75 ± 10.1) and TU212 + CAF/si-nc cells (130 ± 26.3) moved through Matrigel at higher rates than the TU686 + CAF/si-PYROXD1 (46 ± 7.1) and TU212 + CAF/si-PYROXD1 cells (76 ± 13.0, *P < 0.05, **P < 0.01, Fig. 2H–K). Together, these data suggest that downregulation of PYROXD1 expression reduced the proliferation, migration, and invasion of LSCC cells.

### PYROXD1 promotes LSCC cell proliferation, migration, and invasion

**Fig. 3:**
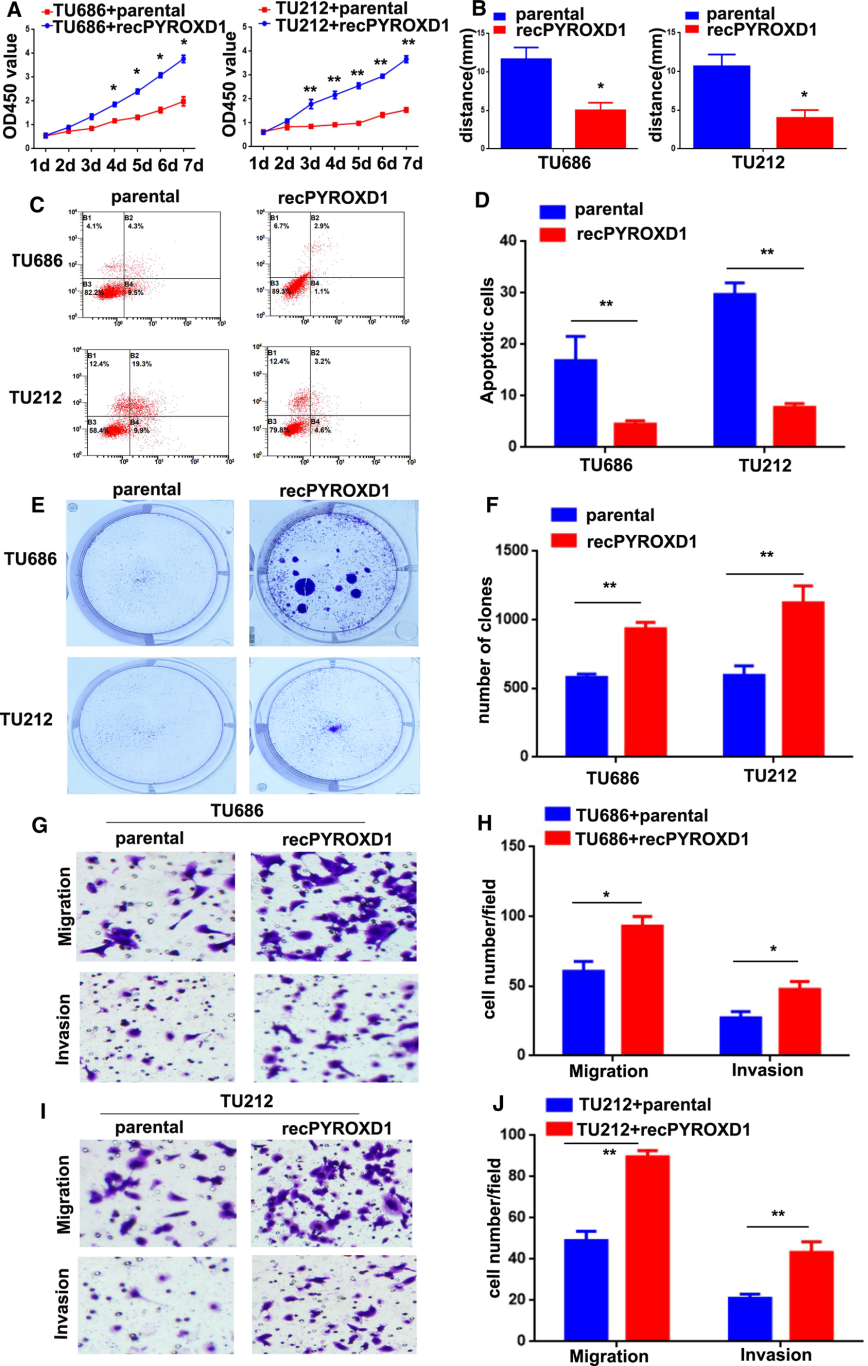
PYROXD1 promotes the proliferation, migration, and invasion of LSCC cells. **A** PYROXD1 increased the proliferation of TU686 + recPYROXD1 and TU212 + recPYROXD1 cells (*P < 0.05, **P < 0.01). **B** PYROXD1 increased the migration of TU686 + recPYROXD1 and TU212 + recPYROXD1 cells. **C**, **D** PYROXD1 decreased the apoptosis of TU686 + recPYROXD1 and TU212 + recPYROXD1 cells (**P < 0.01). **E**, **F** PYROXD1 accelerated the clone formation of TU686 + recPYROXD1 and TU212 + recPYROXD1 cells (**P < 0.01). **G**–**J** PYROXD1 promoted the migration and invasion of TU686 + recPYROXD1 and TU212 + recPYROXD1 cells (*P < 0.05, **P < 0.01). Migratory and invaded Cells were counted in ten randomly selected microscopic fields. Values are represented as mean ± SD of three independent experiments

To investigate the role of ROCK1-induced PYROXD1 expression in activating CAFs to promote LSCC metastasis, TU686 and TU212 cells were treated with purified recombinant PYROXD1 protein (TU686/recPYROXD1 and TU212/recPYROXD1 cells). As shown in Fig. 3A, the proliferation of both the TU686 + recPYROXD1 and TU212 + recPYROXD1 groups was higher than that of the TU686 + parental and TU212 + parental groups (*P < 0.05, **P < 0.01). The wound healing results showed that the TU686 + recPYROXD1 and TU212 + recPYROXD1 cells had greater mobility at 48 h than the TU686 + parental and TU212 + parental cells (Fig. 3B, *p < 0.05). The flow cytometry results showed that the percentage of apoptotic cells was significantly higher in the TU686 + parental (16.8 ± 4.72) and TU212 + parental groups (29.7 ± 2.25) than in the TU686 + recPYROXD1 (4.5 ± 0.65) and TU212 + recPYROXD1 groups (7.8 ± 0.75, **P < 0.01, Fig. 2C, D). Next, colony formation assay was performed to further verify the proliferative effect of PYROXD1, and the results showed that the numbers of colonies in the TU686 + recPYROXD1 (934 ± 46.1) and TU212 + recPYROXD1 groups (1123 ± 125.0) were greater than those in the TU686 + parental (579 ± 25.2) and TU212 + parental groups (595 ± 69.5, **P < 0.01, Fig. 2E, F). Additionally, migration assays indicated that the TU686 + recPYROXD1 (93 ± 7.0) and TU212 + recPYROXD1 cells (89 ± 3.1) migrated at higher rates through Transwell chambers than the TU686 + parental (60 ± 7.0) and TU212 + parental cells (49 ± 4.6, *P < 0.05, **P < 0.01, Fig. 2G–J). Lastly, invasion assays showed that the TU686 + recPYROXD1 (47 ± 5.7) and TU212 + recPYROXD1 cells (43 ± 5.1) invaded through Matrigel at higher rates than the TU686 + parental (27 ± 4.5) and TU212 + parental cells (21 ± 2.0, *P < 0.05, **P < 0.01, Fig. 2G–J). Together, these data suggest that PYROXD1 promoted the proliferation, migration, and invasion of LSCC cells.

### Knockdown of PYROXD1 expression impairs LSCC metastasis in nude mice

**Fig. 4:**
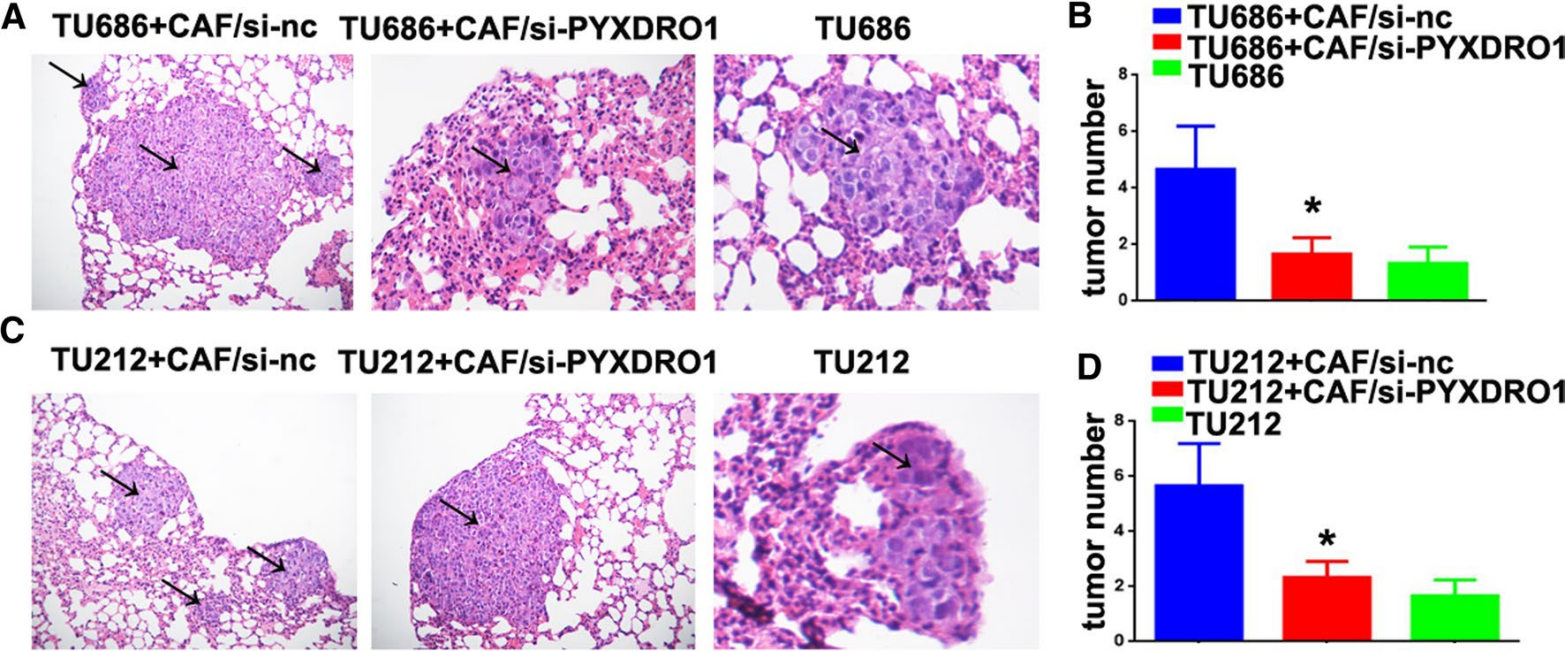
PYROXD1 induces LSCC metastasis in nude mice. **A** TU686 + CAF/si-PYROXD1, TU686 + CAF/si-nc and TU212 + CAF/si-nc and TU686 cells were injected into nude mice. H&E staining of pulmonary nodules (black arrows, ×100). **B** Pulmonary tissue and nodules were analyzed by H&E staining, and pulmonary nodules were observed after 42 days (N = 5/group, *P < 0.05). **C** TU212 + CAF/si-PYROXD1, TU212 + CAF/si-nc and TU212 cells were injected into nude mice. H&E staining of pulmonary nodules (black arrows, ×100). **D** Pulmonary tissues and nodules were analyzed by H&E staining, the nodules were observed after 42 days (N = 5/group, *P < 0.05)

To examine the role of PYROXD1 in the metastasis of LSCC, TU686 + CAF/si-PYROXD1, TU212 + CAF/si-PYROXD1, TU686 + CAF/si-nc and TU212 + CAF/si-nc, TU686 and TU212 cells were injected into nude mice via their tail vein. Six weeks after injection, the TU686 + CAF/si-nc (4.7 ± 1.5) and TU212 + CAF/si-nc (5.7 ± 1.5) groups demonstrated more frequent lung metastases than the TU686 + CAF/si-PYROXD1 (1.7 ± 0.6), TU212 + CAF/si-PYROXD1 (2.3 ± 0.6), TU686 (1.3 ± 0.6) and TU212 groups (1.7 ± 0.6, *P < 0.05, **P < 0.01, Fig. 4A–D). Thus, we conclude that PYROXD1 promoted LSCC metastasis in vivo.

## Discussion

LSCC arises from the larynx epithelium and is one of the most common HNSCCs, with a high rate of metastasis and a poor prognosis [19]. Therefore, elucidating the mechanisms underlying its tumorigenesis, progression and metastasis are of great significance for developing effective clinical prevention programs and new targeted therapies.

In the tumor microenvironment, the cross-talk between tumor cells and tumor stromal cells promotes tumorigenesis and metastasis [20]. The tumor cell matrix contains fibroblasts, endothelial cells and lymphocytes, among which cancer-associated fibroblasts (CAFs) are the main cells in the tumor microenvironment [21]. Studies have shown that CAFs are a heterogeneous cell population and are significantly different from normal fibroblasts (NFs) in phenotype and function. Compared with NFs, the proliferation capacity of activated CAFs is increased [22]. NFs maintain epithelial stability by inhibiting the carcinogenesis and proliferation of adjacent epithelial cells. However, after malignant transformation of the epithelium, CAFs can promote tumor growth by inducing angiogenesis, recruiting bone marrow-derived endothelial progenitor cells and remodeling the extracellular matrix [23]. Functionally, NFs can inhibit the malignant phenotype of epithelial cells and maintain the stability of epithelial cells, while CAFs promote the malignant transformation and growth of epithelial cells by reconstructing the tumor matrix and releasing inflammatory factors such as TGF-β and IGF [24]. Therefore, an in-depth study of CAFs could help elucidate the underlying tumor metastasis and provide a new theoretical basis for interventional strategies for the early diagnosis of tumors. Previous studies have shown that CAFs could significantly promote the metastasis of LSCC [25]. However, the mechanism underlying the effects of CAF activation on LSCC metastasis has still not been thoroughly studied.

In our previous study, we showed that ROCK1 was highly expressed in human LSCC tissues, and high expression of ROCK1 correlated with advanced stage and poor survival of LSCC patients. PYROXD1 is a classic oxidoreductase containing two assumed enzyme domains, including a nitroreductase domain and a pyridine nucleotide disulfide oxidoreductase domain. Studies have shown that PYROXD1 was related to congenital myopathy and regulated the development of muscle tissues [26]. PYROXD1 sinks Murine was reported to reduce the proliferation ability of colorectal cancer cells and could act as a tumor promoter [27]. Amplifications of 12p12.1 (PYROXD1) and KDM5A were correlated with a worse prognosis, which was further validated in a cohort of 506 LUAD patients [28]. Despite accumulating evidence indicating that ROCK1 and PYROXD1 play vital roles in the metastasis of various cancers, the crosstalk between ROCK1 and PYROXD1 in LSCC is unknown.

Previous literature confirmed the overexpression of PYROXD1 in several types of cancers. Here, we described the role of ROCK1-induced PYROXD1 expression in LSCC metastasis. Our data showed that ROCK1 interacted with PYROXD1. LSCC cells cocultured with PYROXD1-knockdown CAFs exhibited reduced proliferation, migration, invasion and metastasis abilities. Conversely, LSCC cells cocultured with PYROXD1-overexpressing CAFs show the opposite results. In conclusion, we showed that ROCK1 could target PYROXD1 to regulate CAF activation and promote LSCC metastasis, indicating the key role of PYROXD1 as an oncogenic protein and revealing a novel molecular mechanism underlying the development and progression of LSCC.

In summary, we demonstrate that the crosstalk between ROCK1 and PYROXD1 regulated CAF activation and promoted LSCC metastasis. Thus, ROCK1 and PYROXD1 molecules could be considered as new therapeutic targets for LSCC. Nonetheless, how ROCK1 regulate PYROXD1 expression? What is the potential mechanism? More experiments should be conveyed in our further study.

## Data Availability

The data and material from the current study are available from the corresponding author upon reasonable request.
